# The use of viral vectors in vaccine development

**DOI:** 10.1038/s41541-022-00503-y

**Published:** 2022-07-04

**Authors:** Tatianna Travieso, Jenny Li, Sneha Mahesh, Juliana Da Fonzeca Redenze E. Mello, Maria Blasi

**Affiliations:** 1 Department of Medicine, Division of Infectious Diseases, Duke University School of Medicine, Durham, NC USA; 2 Duke Human Vaccine Institute, Duke University School of Medicine, Durham, NC USA; 3 Duke University, Durham, NC USA

**Keywords:** DNA vaccines, Translational research

## Abstract

Vaccines represent the single most cost-efficient and equitable way to combat and eradicate infectious diseases. While traditional licensed vaccines consist of either inactivated/attenuated versions of the entire pathogen or subunits of it, most novel experimental vaccines against emerging infectious diseases employ nucleic acids to produce the antigen of interest directly in vivo. These include DNA plasmid vaccines, mRNA vaccines, and recombinant viral vectors. The advantages of using nucleic acid vaccines include their ability to induce durable immune responses, high vaccine stability, and ease of large-scale manufacturing. In this review, we present an overview of pre-clinical and clinical data on recombinant viral vector vaccines and discuss the advantages and limitations of the different viral vector platforms.

## Introduction

Recombinant viral vectors have been used to deliver antigens from specific pathogens for over forty years^1^. The first viral vector expressing a foreign gene was created from the SV40 virus in 1972^2^; since then, a variety of other viruses, including adenoviruses, poxviruses, herpesviruses, vesicular stomatitis virus, and lentiviruses, have been engineered into vaccine vectors to stimulate immune responses against the proteins generated from the encoded transgenes. Viral vectors offer several advantages over traditional subunit vaccines, one of those being that in addition to eliciting potent antibody responses, they also elicit cellular responses that are crucial for the elimination of pathogen-infected cells. Additionally, viral vectors can induce high immunogenicity without the use of an adjuvant, as well as long-lasting immune responses—in some cases after just a single dose^3^. Furthermore, viral vectors can be engineered to deliver vaccine antigens to specific cells or tissues. In an effort to increase their safety profile and to reduce reactogenicity, many viral vectors have been genetically modified to render them replication-deficient. Replication-deficient vectors express the antigen of interest under the control of an exogenous promoter. Antigen expression by vector-transduced cells results in the induction of immune responses, and the durability of antigen expression often correlates with the durability of antibody and T cell responses. Additionally, the presence of the antigen on the surface of the vector particles or inside the virion can also contribute to the induction of an immune response. Although replication-deficient viral vectors are generally safer than replicating vectors, they may require a higher dose or a prime-boost regimen to elicit sufficient immunity^4^, and depending on the specific application, the use of a replicating vector may still be preferred. One advantage of replicating vectors is their mimicking of a natural infection, resulting in the induction of cytokines and co-stimulatory molecules that provide a potent adjuvant effect. When a replication-deficient viral vector does not stimulate the most appropriate responses, the incorporation of an adjuvant may be required to augment the immune response against the encoded antigen(s). Viral vector vaccines can be further optimized to improve transgene expression in target cells, to deliver two antigens simultaneously, to circumvent pre-existing immunity in repeated immunizations, and to reduce potential side effects. Although intramuscular injection is the most common route of immunization to induce systemic immunity, viral vector vaccines can also be administered intranasally, orally, intradermally and via aerosol^5–8^. These alternative routes of immunization can induce immune responses at mucosal sites to prevent or limit respiratory or gastrointestinal infections.

Viral vectors have been used in both pre-clinical and clinical trials as vaccines against a variety of infectious diseases, such as HIV, Malaria, Ebola, and more recently, SARS-CoV-2. They each have their own benefits and risks regarding immunogenicity, safety, and efficacy. Here we describe the different viral vectors that are currently being tested as vaccine platforms in pre-clinical and clinical studies (Table 1) and discuss recent advances in the use of these technologies.

## Adenoviral vector vaccines

**Fig. 1:**
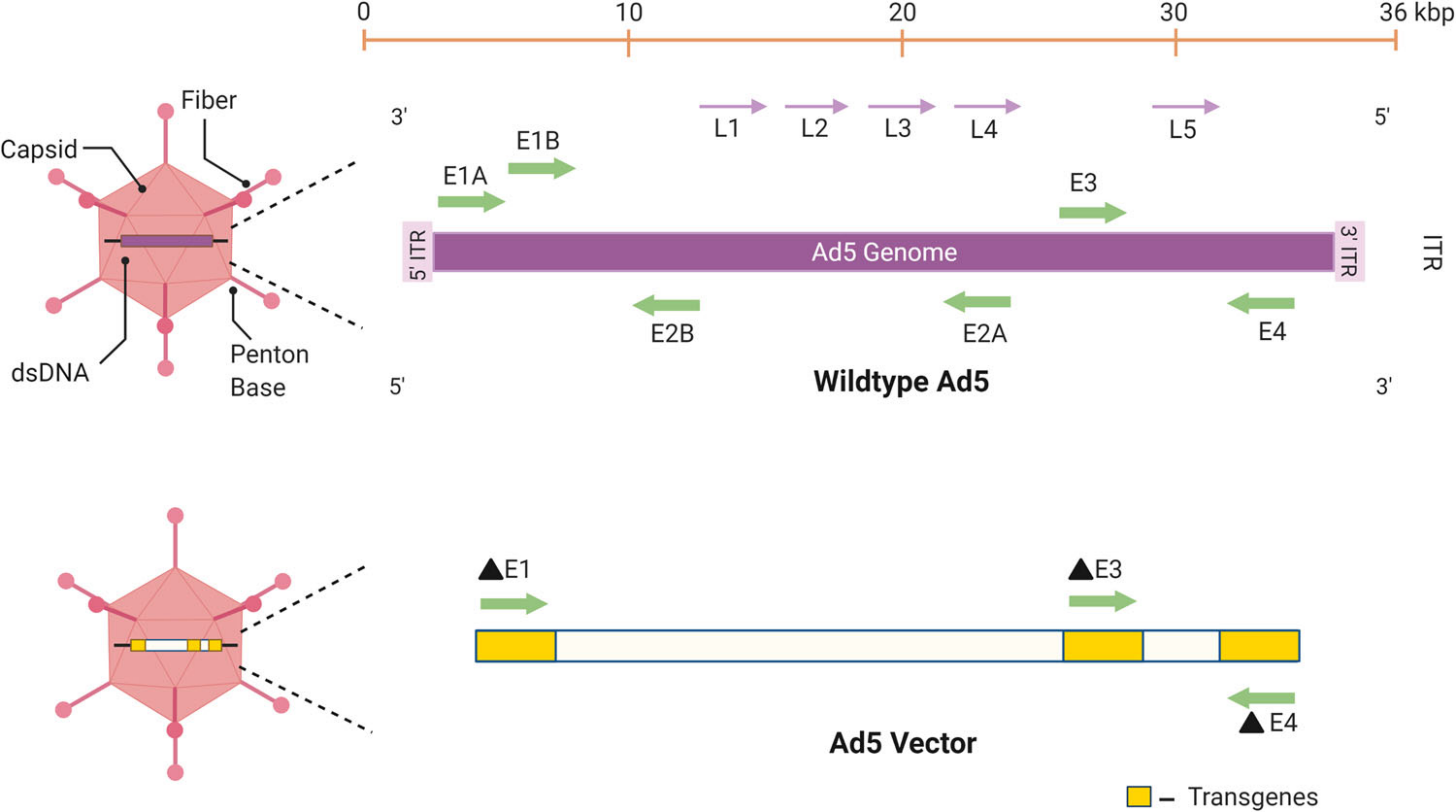
Schematic representation of the Adenovirus 5 linear genome and an Ad5 vector. The adenovirus genome is characterized by inverted terminal repeats (ITR) and several early (E) and late (L) genes. The early genes are responsible for modifying host gene expression to allow for viral protein synthesis and replication. The late genes allow for viral packaging and release. Replication-competent adenoviral vectors contain an intact E1 region and the transgene of choice in the E3 region. Typically, replication-defective adenoviral vectors contain a partial or complete deletion of the E1 region and contain a transgene in either the E1 or E3 region. Deletion of the E4 region may also allow for the insertion of a larger foreign gene.

Several adenoviral vectors are currently being exploited as vaccine delivery systems. Adenoviruses (Ad) are a diverse family of DNA viruses that cause infections in the respiratory, ocular, and gastrointestinal epithelium of their hosts. Their genome is linear and double-stranded with sizes ranging from 26–45 kb^9^ (Fig. 1). Adenoviruses have several advantages as viral vectors for vaccine development, including their relatively low pathogenicity, genetic safety, and the lack of integration in the host genome. Additional attractive properties of Ad vectors for vaccine development include their strong immunogenicity, efficient infection of different cell types, and transgene incorporation capacity^10^. Adenovirus-based vectors can be either replication-competent or replication-defective, depending on whether or not they contain the entire early 1 (E1) region or part of it^10^.

Ad vectors are traditionally manufactured using anchorage-dependent packaging cell lines. The viral vectors are then purified from the lysate using cesium chloride density-gradient centrifugation^10^. For large scale production, the vector is propagated in suspension culture bioreactors using continuous cell lines such as human embryonic retinal cell line (PER.C6). The purification process involves sequential chromatography or chromatography-tandem ultracentrifugation^10^.

## Adenovirus type 5

Adenovirus type 5 (Ad5) has been a common adenoviral vector choice for many viral vector vaccine studies and trials, given its ability to induce both humoral and cell-mediated immune responses^11–15^. However, the use of this vector is hampered by widespread preexisting immunity to Ad5, reaching up to 90% seroprevalence in certain countries^16^, which can negatively affect the vaccine induced immune response^11,14,17,18^. This was particularly evident during the STEP HIV vaccine trial, where vaccination with an Ad5 vector expressing the HIV-1 Gag, Pol, and Nef genes, was associated with increased susceptibility to HIV infection^14,17,19^. As baseline anti-Ad5 neutralizing antibody (nAb) titers increased so did the risk of HIV acquisition, suggesting an effect of preexisting Ad5 immunity on HIV acquisition after vaccination with this vector^11,14,17^. Ad5 nAbs target both the hexon and fiber structural proteins, with nAb responses to hexon as the dominant response after both infection and vaccination^20^. An initial study aimed at understanding the mechanisms of increased susceptibility to HIV acquisition in STEP trial vaccinees suggested that when individuals with pre-existing immunity to Ad5 were vaccinated with the Ad5 vaccine, Ad5 specific CD4 + T-cells would re-activate and serve as targets for HIV-1 infection^17^. This theory was later disproved in a subsequent study that showed no observable differences in Ad5 specific CD4 + T-cells in study subjects who were Ad5 seropositive and those who are Ad5 seronegative^13^. It has also been hypothesized that Ad5 nAbs could increase the risk of HIV acquisition in Ad5 vaccinees through immune complex formation after vaccination, which would lead to alterations in dendritic cell maturation and inflammation^14^. In another study, where male participants were given an Ad5 trivalent HIV-1 vaccine, no difference in HIV progression and set-point viral load were observed between those who received the vaccine and those who received the placebo^15,21^. A more recent study suggested that the elicitation of CD8 + T cells against vaccine-encoded HLA-I adapted HIV-1 epitopes promoted dendritic cell (DC) mediated trans-infection of CD4 + T cells in Ad5 vaccinees^22^. Although there are several hypotheses surrounding the reason for an increase in HIV acquisition observed in HVTN505 participants who were seropositive for Ad5, no study has provided a definitive answer.

Despite these observations, this vector was used in several SARS-CoV-2 vaccine studies. Preliminary immunogenicity data from a randomized, double-blind, placebo-controlled, phase 2 trial of a replication-deficient Ad5 vector expressing the SARS-CoV-2 spike gene has shown that one dose was enough to induce a significant antibody and cytotoxic T-cell responses and that the vaccine was safe overall^12^. A separate study conducted in Russia looked at the safety and efficacy of a SARS-CoV-2 vaccine using rAd5 vector in combination with rAd26^23^. In this phase 3 randomized, double-blind, placebo-controlled trial, the vaccine proved to be safe and showed 91.6% efficacy against SARS-CoV-2 infection^23^. As of February 2022, this vaccine earned full approval from Russia’s Health Ministry after its use under emergency use authorization.

An aerosolized Ad5-based vaccine against SARS-CoV-2 was also tested in a phase 1 clinical trial and shown to be well-tolerated and highly immunogenic^24^. Two doses of the aerosolized formulation induced similar T cell responses and neutralizing antibody titers as one dose of the same vaccine injected intramuscularly, supporting further evaluation of aerosolized vaccines.

Though overall the Ad5 viral vector has proven to be safe and immunogenic in most individuals, the issue of pre-existing immunity to Ad5 led to the development of novel adenoviral vectors based on less prevalent adenovirus serotypes of either human or animal origin^25^, such as Adenovirus type 26 (Ad26) and chimpanzee adenovirus (ChAd).

## Adenovirus type 26

Adenovirus type 26 (Ad26) is less seroprevalent than Ad5, making it an ideal alternative for the development of adenoviral vector based vaccines. Ad26 based vectors have proven to be safe and able to induce both a humoral and cell-mediated response. In a small clinical trial where healthy adults were given a single oral dose of a highly attenuated replication-competent Ad26-HIV-1 vaccine, no antigen specific immune responses were detected, suggesting that the viral vector’s replicative capacity was significantly impaired^26^. A different study using an Ad26 vector expressing mosaic Env/Gag/Pol antigens demonstrated that a mosaic Ad26-based vaccine was both safe and highly immunogenic in both humans and rhesus monkeys^27^. Furthermore, this vaccine protected against repetitive, heterologous, intrarectal SHIV-SF162P3 challenge in rhesus monkeys^27^. These data prompted the Imbokodo phase II/b HIV vaccine clinical trial (ClinicalTrials.gov Identifier: NCT03060629) in a population of young women in sub-Saharan Africa at high risk of acquiring HIV. While the investigational vaccine was found to be safe with no serious adverse events among trial participants, it did not prevent HIV infection and the trial was therefore discontinued. Recently, an Ad26 vector-based vaccine against SARS-CoV-2 has been approved for emergency use to combat the COVID-19 pandemic. This Ad26.COV2.S vaccine is a recombinant, replication-incompetent Ad26 vector encoding a full-length SARS-CoV-2 spike gene^28^. In the randomized, double-blind, placebo-controlled, phase 3 trial, a single dose of the vaccine showed to be safe and effective, with mild adverse events^28^. Although this vaccine proved safe in the phase 2/3 trial, following emergency use authorization by the Food and Drug Administration (FDA) several cases of venous thrombosis and thrombocytopenia were reported in women below 60 years of age between 6 and 15 days post-vaccination^29^. After a temporary pause to allow further investigations into these reported side effects, the use of this vaccine was resumed as its known and potential benefits outweigh its known and potential risks. This vaccine has not yet received full FDA approval.

**Table 1:** Advantages and limitations of viral vector vaccines.

Vector	Vaccine target	Human Trials?	Immune Response	Manufacturing	Pros	Cons
**Ad5** dsDNA non-enveloped	SARS-CoV-2, HIV-1, SIV	Phase III -COVID-19 vaccine approved for human use in some countries	Humoral and cell-mediated	Vector propagation in anchorage-dependent packaging cell lines followed by purification of viruses from the cell lysate by cesium chloride (CsCl) density-gradient centrifugation. For large-scale production, the vector is propagated in suspension culture bioreactors using continuous cell lines.	Induces a robust humoral and cell-mediated immune response	Pre-existing immunity and increasing age can limit induction of immune response; A single injection may not be enough to induce a significant humoral response in individuals over 55; Vaccine-induced thrombosis with thrombocytopenia observed in a minority of vaccinees.
**Ad26** dsDNA non-enveloped	HIV-1, SARS-CoV-2	Phase III -COVID-19 vaccine approved for human use in some countries	Humoral and cell-mediated		Induces a robust humoral and cell-mediated immune response. Low seroprevalence in humans.	Vaccine-induced thrombosis with thrombocytopenia observed in a minority of vaccinees.
**ChAd** dsDNA non-enveloped	Rabies, MERS, SARS-CoV-2	Phase III -COVID-19 vaccine approved for human use in some countries	Humoral and cell-mediated		Induces a robust humoral and cell-mediated immune response. Low seroprevalence in humans.	Vaccine-induced thrombosis with thrombocytopenia observed in a minority of vaccinees.
**AAV** ssDNA non-enveloped	HIV-1, HPV, Influenza	Phase I	Humoral and cell-mediated	HEK 293 T cells transfection with transgene, packaging and helper plasmids. Vector particles can be purified by polyethylene glycol (PEG)-based precipitation, pH-mediated protein removal, and affinity chromatography.	High safety profile, no serious adverse effects, broad tropism, long term gene expression	Requires high dosage due to low immunogenicity; Limited transgene capacity; Pre-existing immunity can limit induction of immune response.
**VSV** ssRNA enveloped	Ebola, MARV, SUDV, BDBV, SARS, Zika Virus, Congo hemorrhagic fever	Phase III - EBOLA vaccine approved for human use	Humoral and cell-mediated	Transfection of mammalian cells with a recombinant VSV plasmid in which the G gene is replaced with the transgene of choice. Virus is then propagated in Vero or HEK-293 cells. For large-scale production, an ion-exchange column is used to purify the virus that can be further concentrated by tangential flow ultrafiltration.	Non-human pathogen; Vaccine can be mucosally administrated.	Mild symptoms post-vaccination due to viral shedding/viremia; Potential for neurovirulence in young populations.
**IDLV** ssRNA enveloped	Malaria, HIV-1, Cancer, Zika Virus, SARS-CoV-2	Phase II (Cancer)	Humoral and cell-mediated	HEK 293 T cells transfection using transfer, envelope, and packaging plasmids of choice. Purify culture supernatants by ultracentrifugation on a sucrose cushion. For large-scale production, the vector can be propagated in suspension culture bioreactors using a stable packaging cell line.	Low to no pre-existing immunity; Absence of integration mitigates risk of insertional mutagenesis; High immunogenicity without any adjuvant; High durability of immune responses due to antigen persistence at injection site.	Safety concerns given lentivirus origin; Potential batch to batch variation in large-scale manufacturing process for clinical products.
**POX** dsDNA outer membrane	HIV-1, TB, Malaria	Phase III	Humoral and cell-mediated	Recombinant vector is generated via homologous recombination in poxvirus infected cells. The recombinant vector can be further propagated in susceptible cells and vector particles are purified by ultracentrifugation on a sucrose cushion. For large-scale production ion exchange and gel filtration chromatography are used to purify poxvirus vectors.	Well-tolerated with no serious adverse events; Large cargo capacity for heterologous genes.	Low immunogenicity when administered alone.

## Chimpanzee adenovirus

Adenoviral vectors based on chimpanzee adenovirus (ChAd) serotypes have been developed and tested against several pathogens including rabies, MERS, and more recently, SARS-CoV-2^30–32^. In a study by Zhou et al., oral/intranasal immunization with a ChAd-vector based anti-rabies vaccine (AdC68rab.gp) induced sustained mucosal antibody response and protection against intranasal virus challenge^31^. ChAd has gained momentum among viral vector vaccines, with the ChAdOx1 nCov-19 vaccine against SARS-CoV-2 rapidly entering a phase III clinical trial in 2020 and receiving subsequent approval for emergency use authorization in several countries^30^. In the phase 1/2 trial, this replication-deficient chimp adenovirus vector expressing the spike protein of SARS-CoV-2 was administered once to 543 participants via an intramuscular deltoid injection^30^. A smaller subset of 10 individuals received a booster vaccine on day 28. Neutralizing antibodies developed in 91% of study participants by day 28 and in all the participants after the boost. In the phase 2/3 trial, the vector proved to be safe and immunogenic for a wider age range^33^. Severe adverse events including transverse myelitis were reported in 1 of 127 trial participants after the boost vaccination. ChAdOx1 nCov-19 appeared to be better tolerated by older adults than young adults, though immunogenicity remained consistent among age groups^33^. Although this vaccine proved safe in the phase 2/3 trial, following emergency use authorization several additional cases of venous thrombosis and thrombocytopenia were reported in 34 to 54 years old vaccinees between 7 and 14 days after the first vaccine dose^34,35^. Shultz et al. analyzed five patients who presented with venous thrombosis and thrombocytopenia 7 to 10 days after receiving the first dose of the ChAdOx1 nCoV-19 vaccine and found that all the patients had high levels of antibodies to platelet factor 4 (PF4)–polyanion complexes^35^. The exact mechanism(s) by which adenoviral vector vaccines trigger the development of anti-PF4 antibodies in a small percentage of vaccinated individuals is unknown. Preliminary hypotheses include the possibility that components of the vaccine bind to PF4 and generate a neoantigen^36^. Indeed, in a recent elegant study by Baker et al., the authors demonstrated that PF4 is capable of forming stable complexes with several adenoviruses including ChAdOx1, Ad 5, and Ad26^37^. These Ad/PF4 complexes could then induce anti-PF4 autoantibodies. Since PF4 contacts residues in the hypervariable regions (HVR) on the surface of these vectors, substitutions of the amino acids important for this interaction could lead to the development of safer adenoviral vectors.

## Adeno-associated viral (AAV) vector vaccines

**Fig. 2:**
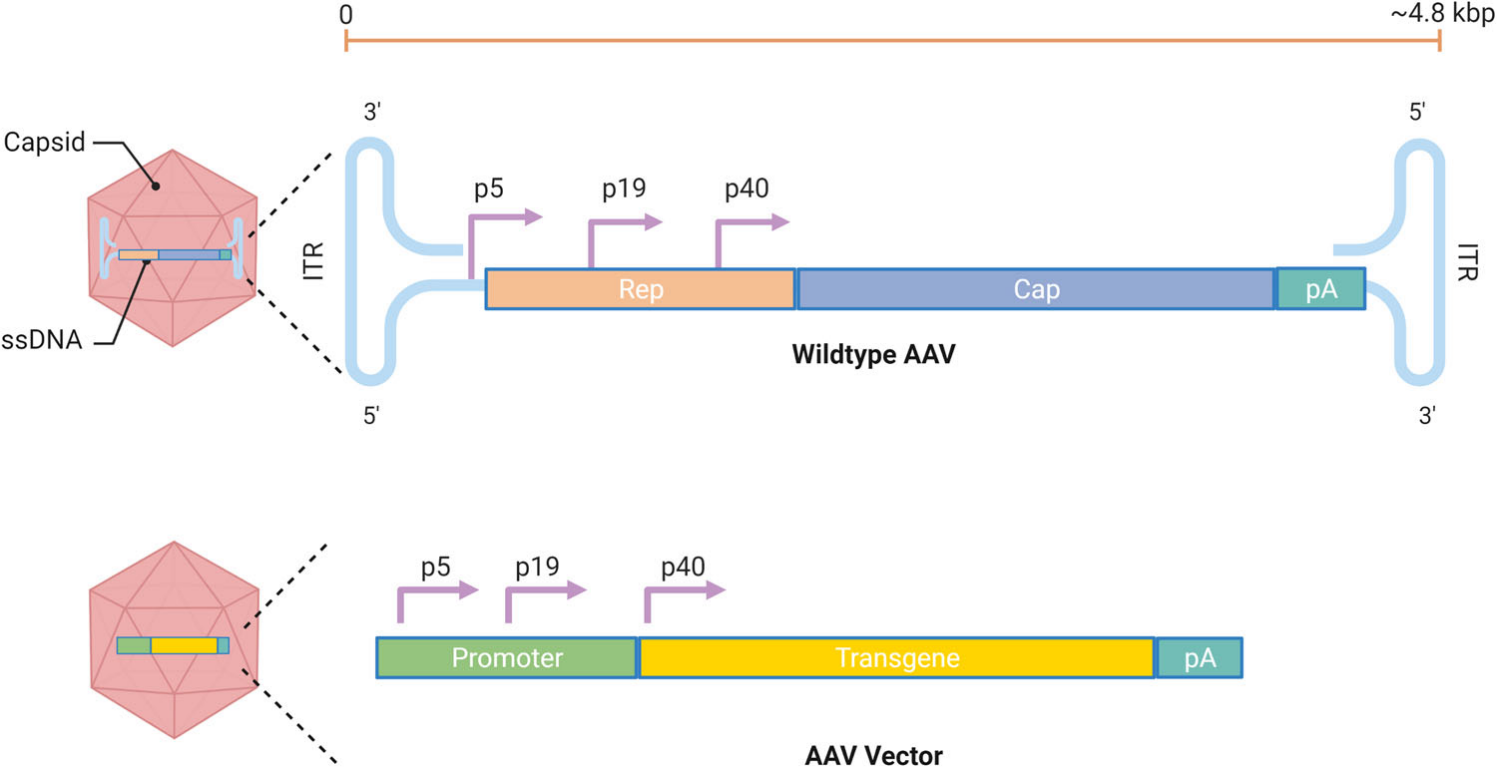
Schematic representation of the AAV genome and an AAV vector. AAVs are small (~25 nm), non-enveloped viruses and have a 4.8-kb, single-stranded, linear DNA (ssDNA) genome encoding four open reading frames: rep encodes the four genes required for genome replication (Rep78, Rep68, Rep52, and Rep40), cap encodes the structural proteins of the viral capsid (VP1, VP2, and VP3). When the viral vector is used in vaccinations, the transgene of choice is placed in the promoter at the p40 location.

Adeno-associated viruses belong to the *Parvoviridae* family. They are non-enveloped viruses with a genome consisting of 4.8 kb linear, single-stranded DNA (Fig. 2). AAV vectors are the most popular choice for gene therapy and delivery of therapeutic antibodies thanks to their relatively low immunogenicity, high safety profile, broad tropism, and their tendency to maintain long-term gene expression^38^. AVV vectors are produced through transfection of human embryonic kidney (HEK) 293 T cells with transgene, packaging and helper plasmids^39^. Vector particles can be purified by polyethylene glycol (PEG)-based precipitation, pH-mediated protein removal, and affinity chromatography^40^. While AAV vectors have been primarily employed to treat eye and muscle diseases^41,42^, their use as vaccine vectors has increased in recent years to treat and prevent infectious diseases, such as HIV, HPV, and influenza^38^. Several studies point out that AAV vector vaccines can induce strong and lasting antibody responses after only one dose and without the need for an adjuvant^43–45^. In some instances, they have also shown to produce a higher or more sustained antibody response relative to other vaccination strategies, such as DNA, recombinant proteins, inactivated viruses, or virus-like particles (VLPs)^44,46–50^. However, AAV vectors are considered to possess a low immunogenic profile when compared to other viral vectors. The isolation of multiple AAV serotypes and capsid variants offers the possibility to develop prime/boost strategies where the AAV capsid can be switched to avoid the anti-capsid neutralizing antibody response induced after the prime. The major drawbacks for AAV vectors include the limited transgene capacity and broad pre-existing immunity in humans. Strategies to improve AAV immunogenicity and to circumvent pre-existing immunity are currently under evaluation. Recombinant AAV vectors are, however, the vector of choice for passive immunizations to deliver monoclonal antibodies and derivatives of immunoglobulins given their ability to maintain long-term expression and high levels of these therapeutics^51–53^.

## Vesicular stomatitis vector vaccines

**Fig. 3:**
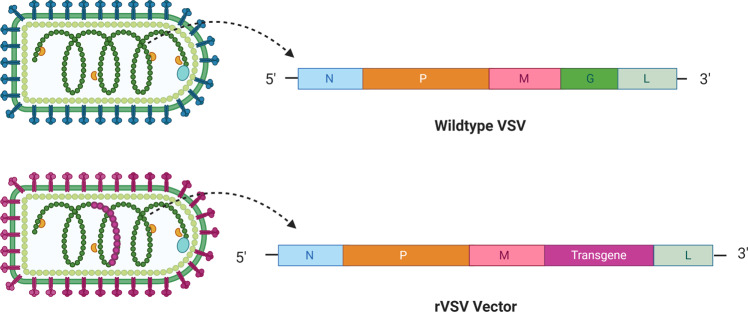
Schematic representation of the wildtype VSV and an rVSV vector. The wild type virus is a negative-sense RNA virus that encodes the nucleoprotein (N), phosphoprotein (P), matrix (M), glycoprotein (G), and RNA-dependent RNA polymerase (L) proteins. When the viral vector is used in vaccinations, the G gene is replaced with the transgene of choice.

The vesicular stomatitis virus, or VSV, belongs to the *Rhabdoviridae* family and is a single-stranded, negative-sense RNA virus characterized by a bullet shape (Fig. 3). VSV commonly infects cattle, horses, pigs, and goats causing lesions along the gums, lips, tongue, nostrils, and udders, sometimes accompanied by a fever and excessive salivation^54^. Transmission to humans may occur, but it most often results in asymptomatic infections. The viral genome is non-segmented and encodes the N, P, M, G, and L proteins (Fig. 3). The G protein, or glycoprotein, mediates the attachment of the viral particle to host cells. VSV represents a valuable vaccine platform thanks to its ability to replicate at high titers, its low seroprevalence, and little pre-existing immunity in humans. VSV-based vaccines are produced through transfection of mammalian cells including baby hamster kidney cells stably expressing T7 polymerase (BHK-T7) or HEK293T with a recombinant VSV plasmid expressing the transgene of interest in place of the virus’ G gene^55^ (Fig. 3). The recombinant virus is further propagated by infecting the African green monkey kidney cell line Vero or HEK-293 cells. For large scale production of rVSV, an ion exchange column is used to purify the virus that is further concentrated by tangential flow ultrafiltration^56^.

The VSV-based recombinant viral vector (rVSV) has demonstrated safety and immunogenicity in pre-clinical and clinical trials^57–60^ and a VSV-based vaccine against Ebola (rVSV-ZEBOV) has recently been approved for use in humans^61^. The rVSV-ZEBOV vaccine lacks the G gene of VSV, which is responsible for the wildtype virus’ ability to infect a broad range of cell types^62^, and expresses the Ebola glycoprotein (GP) in its place. The clinical trials involving the rVSV-ZEBOV vaccine wrapped up at the end of 2018, and the results proved that this vaccine was highly effective across diverse populations. Interestingly, results from both pre-clinical^57^ and clinical studies^63^ demonstrated induction of high levels of neutralizing IgM, suggesting that this antibody isotype may play an important role in vaccine-induced immunity against EBOV. The rVSV platform is being evaluated to develop vaccines against other filoviruses, including the Sudan virus (SUDV)^64^ and Bundibugyo virus (BDBV)^65^, as well as coronaviruses^66,67^, Zika Virus^68^, and Congo hemorrhagic fever, and all these studies have shown promising results.

## Integrase defective lentiviral vector vaccines

**Fig. 4:**
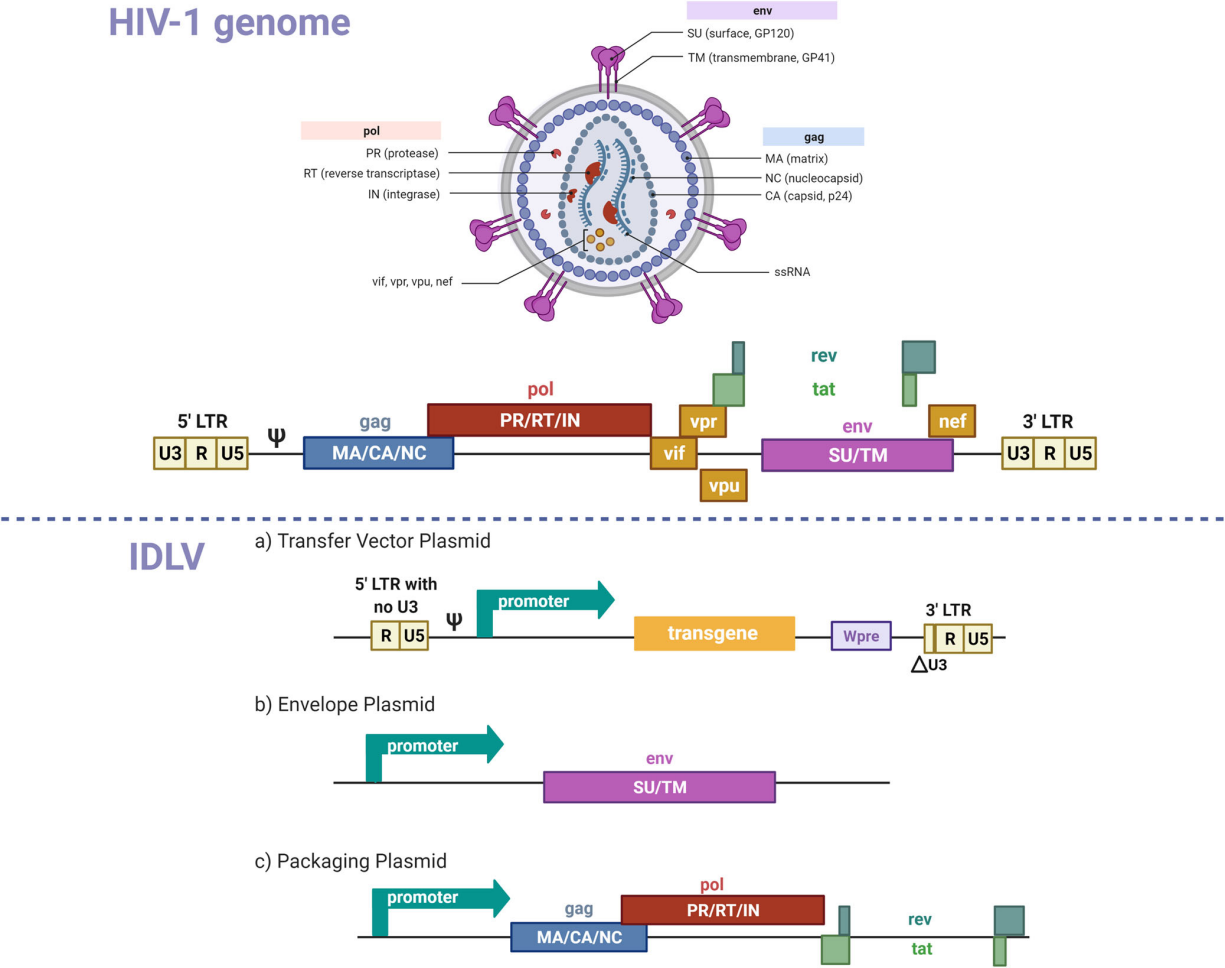
A schematic overview of the lentiviral vector system. The HIV-1 virion and genome are included in the top panel for reference. **a Transfer Vector Plasmid**: this plasmid combines the 5’ and 3’ long terminal repeats (LTRs), and psi component of the HIV-1 genome, along with a promoter, transgene, and the woodchuck hepatitis virus regulatory element (Wpre). The full deletion of the U3 (unique 3’ end) region in the 5’ LTR and partial deletion of the U3 region in the 3’ LTR renders the vector self-inactivating (SIN). **b Envelope plasmid:** this plasmid contains a promoter to drive the expression of the VSV-G envelope protein (env) used to pseudotype lentiviral vector particles. **c Packaging plasmid:** This plasmid contains a promoter to drive expression of the group specific antigen (gag), DNA polymerase (pol), rev, and trans-activator of transcription (tat) elements of the HIV-1/SIV genome.

Integrase-defective lentiviral vectors (IDLVs), represent a promising vaccine platform thanks to their ability to induce high magnitude and very durable immune responses even after a single immunization^29^. IDLVs can be derived from either HIV or SIV by splitting the viral genome into different plasmids and by mutating the long terminal repeats (LTRs), the packaging signal, and the integrase gene to render them replication-deficient and non-integrating. IDLVs are produced by co-transfection of HEK 293T cells with the transfer, envelope, and packaging plasmids of choice (Fig. 4). Culture supernatants containing the vector particles are purified and concentrated by ultracentrifugation on a 20% sucrose cushion^69^. For large scale production the vector can be propagated in suspension culture bioreactors using a stable packaging cell line^69^. IDLV persists in the target cell in an episomal form and can produce the encoded protein for the lifetime of the cell. The non-integrating phenotype of IDLVs is an important safety feature due to the potential for insertional mutagenesis with integrating vectors^70^. IDLV-based vaccines against HIV, Zika, Malaria, and SARS-CoV-2 have been used in several pre-clinical studies in mice and rhesus monkeys, and demonstrated high immunogenicity and durable, protective immune responses^71–78^. IDLV is an attractive delivery platform due to little pre-existing immunity in humans that can be coupled with a vesicular stomatitis virus G envelope glycoprotein (VSV.G) serotype exchange strategy to reduce anti-vector immunity for repeated IDLV injections^72,73,79^. Our group recently showed that following sequential vaccinations of rhesus monkeys with an IDLV-expressing a series of HIV-1 envelope proteins, immune responses were strongly boosted after each of the six vaccinations performed over 3 years with no evidence of tolerance induction^79^. In addition to being highly immunogenic and inducing durable responses, IDLV also demonstrated an excellent safety profile. In extensive safety studies by our group and others, no replication-competent lentiviruses (RCL) have been detected in IDLV-injected animals^72,74,79^. Long-term immunity induced by IDLV may be ascribed to the persistence of IDLV in vivo. Indeed, retro-transcribed vector DNA could still be detected in the muscle of immunized NHPs at 6-months post-injection^72^ and, in mice, transgene expression was confirmed by both IHC and RT-PCR up to 3-months post-injection in muscle tissue^72,80^, suggesting that IDLV can provide persistent transgene expression.

Other studies have also shown the efficacy of IDLV as a therapeutic vaccine as opposed to strictly prophylactic. Therapeutic immunization with IDLV expressing SIV-Gag increased CD8 + T cell responses and induce prolonged virus control in chronically SHIV-infected rhesus monkeys^73^. In this study, an inverse correlation between the magnitude of T cell responses and viral load was observed, and virus control was sustained for over 20 weeks post-IDLV-SIV-Gag injection^73^. These studies emphasized the powerful potential IDLVs have as both preventive and therapeutic vaccines.

## Poxvirus vector vaccines

Poxviruses are a large family of complex, enveloped viruses, including Vaccinia virus (VV) and Variola virus, the causative agent of smallpox. Poxviruses have a double-stranded DNA genome and are unusual as they can replicate in the cytoplasm using viral polymerases to undergo replication and transcription^81^. Poxvirus-based vaccines have been used against various infectious diseases, such as HIV-1, tuberculosis and malaria. One advantage of using poxviral vectors is their cargo capacity for heterologous genes of around 25 kb which is far larger than that of other vectors^81^. This makes poxviral vectors ideal candidates for the generation of multi-antigen vaccines against different pathogens. Poxvirus vectors are usually produced via homologous recombination in poxvirus infected cells. Commonly used cell lines include the kidney epithelial cells CV-1, Vero, and BSC-40 cells^82^. Poxvirus infected cells are transfected with the recombinant transfer plasmid and the produced recombinant vector can be further propagated in susceptible cells. Vector particles are purified by ultracentrifugation on a sucrose cushion. For large scale production, ion exchange and gel filtration chromatography are used to purify poxvirus vectors^83^.

**Fig. 5:**
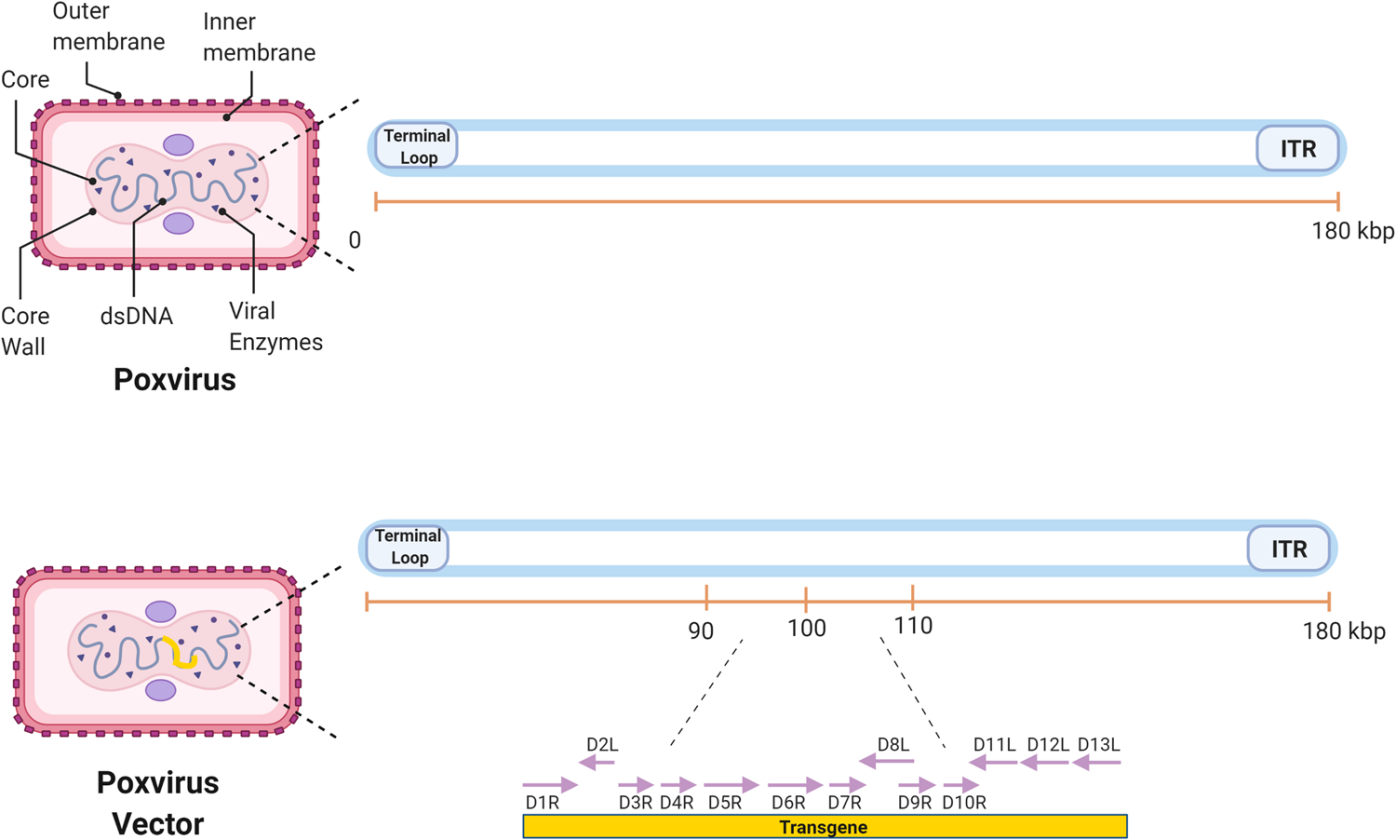
Schematic representation of a poxvirus genome flanked by one origin for DNA replication and one terminal loop. The central region of the genome contains a conserved series of genes needed for viral replication. The two flanking regions code for several proteins that help determine virulence. When used as a viral vector in vaccinations, the D1–13 transcription units (indicated between the dotted lines) are replaced by the transgene of choice.

Poxvirus vectors have shown to be highly immunogenic and able to induce robust immune responses. These viral vectors are either naturally replication-deficient in humans due to host-range restriction or can be rendered replication-deficient by serial passaging in avian cells, as in the case of the modified vaccinia Ankara (MVA), which leads to the loss of genes required for infection of human cells^84,85^ (Fig. 5). A type of poxvirus that has shown promise as a vaccine delivery platform is the modified vaccinia Ankara (MVA) vector. A study of an MVA-vectored HIV-1 mosaic bivalent vaccine showed that a humoral and cell-mediated immune response could be elicited in humans. The vaccine was also safe, well-tolerated, and immunogenic^86^. Another replication-deficient VV strain being used in vaccine studies is the New York Vaccina Virus (NYVAC). NYVAC recombinant vectors with HIV gag-pol-nef antigens and viral envelope genes have proven to boost CD4 + and CD8 + T-cell responses. The same study showed that while an MVA/NYVAC vector combination gave a broad immune response, a combination of DNA/NYVAC gave a stronger immune response^87^. These results are further supported by a recent study that looked at the efficacy of a DNA vector, NYVAC vector, and a combination of the two. The study showed that a combination of the two vectors led to an earlier and more potent antibody response^88^. Another study looked at ways to improve the existing NYVAC- HIV vector vaccine by making it replication-competent, which increased its ability to induce a robust T-cell response^89^. One of the most successful HIV vaccine trials to date was the RV144 trial using a replication-deficient poxvirus vector^90^. ALVAC-HIV was a recombinant canarypox vector that was used alongside a recombinant glycoprotein 120 subunit vaccine. It was given to over 16,000 participants aged 18−30 in Thailand^90,91^. The vaccine demonstrated 31.4% efficacy in reducing the risk of HIV acquisition^91^, although it did not affect viral load or CD4 + counts in people who became infected. Immune correlate analysis on RV144 vaccinees demonstrated that vaccinees with the highest IgG-binding antibodies against the variable loops 1 and 2 (V1V2) of the HIV-1 Env, were more likely to be protected than those with low titers^92^. However, subsequent trials conducted in Thailand^93^ and South Africa^94^ using a similar vaccine failed to show any efficacy^95^.

## Heterologous viral vectors vaccines

Heterologous viral vector vaccines (HVVV) consist of a combination of two or more vectors encoding either the same or different antigens that can be administered together or in prime/boost regimens. HVVV immunizations can be advantageous to induce higher magnitude and more durable immune responses compared to homologous vector vaccination. Immunization of rhesus monkeys with a combination of VSV, vaccinia virus (VV), and Ad5- vectors expressing SIVmac239 Gag, in concert with 3M-052 (TLR7/8 ligand) adjuvanted protein resulted in protection from viral challenge in 67% of the vaccinated animals, which was superior to the protection observed in animals vaccinated with adjuvanted protein alone (53%)^96^. Interestingly, the study found that HVV vaccination induced potent anti-Gag CD8 + T cell responses that reduced the threshold of neutralizing antibodies required to confer durable protection^96^.

The HVVV strategy has recently been used clinically as a novel Ebola vaccine candidate, using a recombinant replication-deficient adenovirus chimpanzee serotype 3 (ChAd3) vector expressing wild-type Ebola glycoprotein (ChAd3-EBO-Z) vector as a prime and an MVA vector expressing the same antigen (MVA-EBO-Z) as a boost^97^. The phase 1a study was conducted in 40 healthy UK volunteers to assess the safety and immunogenicity of MVA-EBO-Z alone and the heterologous prime-boost regimen of ChAd3- EBO-Z followed by MVA-EBO-Z. The phase 1b trial was conducted in 40 Senegalese adults with the same age range (18–50 y.o.) receiving the heterologous vaccine. The humoral response post-immunization was significantly lower in Senegalese volunteers than in the UK cohort, and was likely due to an increased burden of pathogen exposure, genetic differences, microflora composition, and nutritional status. Antibody titers and cellular immune responses in the vaccinees that received the MVA-only vaccine were significantly lower than those in the groups that received the heterologous ChAd3-EBO-Z prime and MVA-EBO-Z boost, supporting the use of heterologous vaccines to increase the magnitude of immune responses.

Depending on the combination of viral vectors used in the heterologous vaccination, different types of immune responses can be induced. This was evident during a study that evaluated the immunogenicity and protective efficacy of prime-boost immunization strategies against malaria in humans^98^. In this study, 3 vaccine platforms including a plasmid DNA, an MVA vector, and an attenuated strain of fowlpox FP9 were used in different prime/boost combinations. Study results demonstrated that the different vectors need to be used in a specific order to induce an optimal IFN-gamma response. In particular, a DNA prime followed by an MVA boost and an FP9 prime followed by an MVA boost were the most immunogenic and induced an IFN-gamma response of broad specificity that was cross-reactive against two *P. falciparum* strains^98^.

In addition to HVVV strategies, viral vectors have also been used in prime-boost strategies with DNA or mRNA based vaccines^98,99^. Notably, the halt of the adenovirus-based ChAdOx1 COVID-19 vaccine due to the rare adverse effects described above, led to booster vaccinations of partially vaccinated individuals who had received a single dose of ChAdOx1 with a second dose of the Pfizer BNT162b2 mRNA vaccine or the Moderna mRNA-1273 vaccine. When comparing the protection conferred by homologous ChAdOx1 vaccination to that of heterologous vaccination with ChAdOx1 and mRNA, several studies found the latter to be superior^100,101^. Barros-Martins et al. found that the ChAd/BNT regimen stimulated a higher level of anti-spike IgG and IgA when compared to the ChAd/ChAd regimen^100^. Neutralizing antibody titers against several SARS-CoV-2 variants of concern [(Alpha, B.1.1.7), (Beta, B.1.351), and Gamma (P.1)] were also 20–60-fold higher in the ChAd/BNT group as compared to the ChAd/ChAd group. Similarly, Schmidt et al. found that ChAd/mRNA vaccination (for both BNT162b2 and mRNA-1273) led to higher titers of anti-spike and anti-receptor binding domain IgG antibodies than ChAd/ChAd vaccination^101^. When comparing the heterologous vector/mRNA regimens to homologous mRNA regimens, heterologous vaccination led to higher levels of spike-specific CD8^+^ T cells. These studies support the exploration of heterologous vaccination strategies using different platforms given the potentially improved immunogenicity of the vector/mRNA regimen.

## Insect-specific viruses viral vectors vaccines

Recent years have seen the advent of viral vectors based on insect-specific viruses (ISV). Several of these viruses belong to viral families associated with animal arbovirus pathogens, such as *Flaviviridae*, *Togaviridae* and *Phenuiviridaeare*, but their replication is typically restricted to insects only^102^. These ISVs have been engineered to produce chimeric particles vaccines expressing chimeric antigens for a range of vertebrate-infecting viruses, including *Flaviviruses* and *Togaviruses*^103,104^. While these chimeric vaccine viruses are replication-deficient in vertebrate cells, they grow to high titers in insect cells and their particles are antigenically and structurally indistinguishable from the corresponding wild type viral pathogens. A chimeric vaccine between two mosquito borne alpha viruses, the ISV Eilat virus (EILV) and the human pathogenic chikungunya virus (CHIKV), induced both antibody and T cell responses in mice and protected NHPs from CHIKV after a single dose^104^. Differently from the mouse study, no significant differences in T cell responses were detected between vaccinated and unvaccinated NHPs, supporting the role of nAbs in protection from viral challenge.

## Conclusions

Viral vectors have been around for over forty years and many of them have been used and are currently being used as vaccines against infectious diseases as described here. The SARS-CoV-2 pandemic has pushed forward the development of viral vector vaccines and highlighted both the strengths and limitations of some of those platforms. Further engineering of those vectors will be required to improve reactogenicity, efficacy, and vector dose.
